# Analysis of the bond interface between self-adhesive resin cement to eroded dentin *in vitro*

**DOI:** 10.1371/journal.pone.0208024

**Published:** 2018-11-26

**Authors:** Mariana Dias Moda, Ticiane Cestari Fagundes, André Luiz Fraga Briso, Paulo Henrique dos Santos

**Affiliations:** 1 Department of Restorative Dentistry, Araçatuba Dental School, UNESP—São Paulo State University, Araçatuba, SP, Brazil; 2 Department of Dental Materials and Prosthodontics, Araçatuba Dental School, UNESP–São Paulo State University, Araçatuba, SP, Brazil; Institute of Materials Science, GERMANY

## Abstract

The purpose of this study was to evaluate the bonding interface between a self-adhesive resin cement to *in vitro* eroded dentin. Seventy-two third molars were used and divided into two groups: sound dentin and *in vitro* eroded dentin. The *in vitro* erosion was performed following a demineralization protocol, in which the specimens were immersed in a demineralizing solution for 2 minutes per cycle and remineralizing solution for 10 minutes per cycle for 9 days. Both groups were submitted to four dentin surface treatments: control group (without any treatment), 2% chlorhexidine, 20% polyacrylic acid, and 0.1 M EDTA (n = 9). Blocks of resin-based composite were bonded with RelyX U200 self-adhesive resin cement applied on the pretreated dentin surfaces. The teeth were sectioned into beams (1mm^2^) and submitted to microtensile bond strength testing to evaluate the bond strength of self-adhesive resin cement to dentin after 24 hours and 8 months of immersion in artificial saliva. Three specimens of each group were longitudinally cut and evaluated using confocal laser scanning microscopy to analyze the dentin/cement interface. Eroded dentin showed higher bond strength values when compared to sound dentin for the 2% chlorhexidine group (p = 0.03), 24 hours after adhesion. When considering eroded dentin, the 0.1M EDTA group showed higher bond strength values with a statistically significant difference only for the control group (p = 0.002). After 8 months of storage, the present results showed that there was no statistically significant difference between the two substrates for all experimental groups (p>0.05). Analysis of the microscopy confocal showed different types of treatments performed on dentin generally increased tags formation when compared to the control group. The eroded dentin showed a significant increase in density and depth of resinous tags when compared to sound dentin. The storage of samples for 8 months seems to have not caused significant degradation of the adhesive interface.

## Introduction

The focus of restorative dentistry has changed over time. Until recently, caries disease was the main restorative concern; however, non-carious cervical lesions have increased in clinical relevance due to the significant incidence of the cases [1], especially for erosive lesions. This increase is due to the natural aging of a large part of the population, which allows teeth to be maintained longer in the mouth due to improved oral health, and increased consumption of acidic foods [2,3].

Dental erosion may be defined as an irreversible loss of dental tissue when exposed to a chemical process without bacterial involvement [4]. It has a multifactorial etiology and could be intrinsic or extrinsic. The intrinsic lesions occur when tissue loss is associated with gastroesophageal disorders, such as recurrent vomiting in individuals with bulimia and anorexia, usually related to psychosomatic diseases [5], which occurs frequently in Occidental society, especially among women between 16 and 35 years [6]. The extrinsic lesions occur by ingestion of beverages and acidic foods, in addition to the use of hygiene products and acidic drugs [7]. It is believed that the erosive potential of acidic beverages is not only linked to its pH [7], but it is also associated with the mineral content of dental tissues, titratable acidity and calcium chelating properties [2], and alcohol consumption [5]. Salivary function is extremely relevant in the erosion dynamic, since its mineral composition and capacity to neutralize acids may be responsible for accentuating or minimizing these lesions [5,8]. The erosive process could also be associated with abrasive forces [9], in which the susceptibility to abrasion can be increased when the tooth is exposed to acids that cause surface softening [10]. Therefore, the presence of abrasive substances in a dentifrice, as well as the diameter of the toothbrush bristles, could affect the erosion process [10,11].

Erosion promotes demineralization and exposure of organic structures, mainly formed by collagen [12]. The degree of degradation can be determined by hydroxyproline analysis, the main aminoacidic in collagen [6]. Furthermore, some proteolytic enzymes, such as pepsin and trypsin, constituents in the digestive tract and frequently found with oral microbiota, can remove this organic matrix through a biochemical process, accentuating the dentin erosive process [13].

Thus, several *in vitro* studies have been performed to simulate and understand this erosive dynamic [1,6,7,13,14]. A very common study method uses citric acid, which is present in beverages, in the dissolution process of enamel and dentin [14]. Citric acid is used because it has a great erosive potential, due to its ability to chelate calcium from dental tissues and saliva [15]. However, Caneppele et al. [7] showed greater susceptibility of dental tissues to erosion when concentrations of calcium and phosphate were low, making the critical pH more susceptible to the erosive process with different beverages, such as wine and energy drinks. In addition, the low degree of saturation of the hydroxyapatite and fluorapatite can lead to structural defects of the tissues, making them more prone to erosion [7]. Another way to achieve *in vitro* erosion is the use of hydrochloric acid, pepsin, and trypsin; all of which are present in the gastrointestinal tract, simulating conditions of constant vomiting in patients with eating disorders, gastroesophageal disease, anorexia, or bulimia. The combination of pepsin and trypsin is intended to accentuate erosion wear [6, 13,16].

The cementation procedure of indirect restorations is very often performed without preparation or any previous treatment, which can include sound or previously eroded dental tissues. The cementation process of aesthetic restorations has been modified in recent years; the introduction of self-adhesive resin cements has provided better time optimization and reduced the number of clinical steps [17,18]. These cements do not require prior acid etching of dental tissues or the application of adhesive systems [18]. They have phosphoric acid and resinous monomers in their organic matrix, which superficially demineralize enamel and dentin, partially removing the smear layer and providing chemical and micromechanical retention [19]. Chemical bonding is achieved through the linkage of resin monomers to calcium ions in hydroxyapatite [17,19]. However, due to the limited capacity of the monomers to totally dissolve the smear layer, the dentin-cement interface exhibits weak bonding due to the formation of short resin tags [20,21].

Some substances have been studied to optimize the bonding interface over time. Chlorhexidine, although frequently utilized in the disinfection of root canals, has been used as a dentin surface treatment [22,23]. It has an antibacterial effect as an inhibitor of metalloproteinases (MMPs), specifically MMPs 2, 8, and 9 [24,25], as well as some proteins, such as the cysteine cathepsin [26]. MMPs are enzymes that act in the degradation of the collagen network, which can negatively interfere with bond strength [19]. Di Hipólito et al. [27] showed that the use of chlorhexidine, at concentrations of 0.2% and 2%, decreases the bond strength of the resin cement to dentin, which can be explained by its capacity to modify the permeability of the smear layer. Another substance that has been used as a surface treatment to reinforce the bonding of resin cements to dentin is polyacrylic acid, which is considered a weak acid, by promoting mild demineralization of the inorganic layer [21,23]. The ethylene diamine tetra acetic acid (EDTA), through calcium chelation, would promote exposure of the collagen fibers with greater penetration of a resin cement into demineralized dentin [22]. However, the use of this substance before using self-adhesive resin cements that depend on calcium in the dentin surface needs to be better elucidated.

The lack of studies that analyze the behavior of the adhesive materials to eroded dentin submitted to pre-treatments, such as EDTA, means that the results could contribute to the development of a new clinical protocol for this substrate. In this manner, studies are necessary to provide a scientific basis for the best clinical practice.

The purpose of this study was to evaluate the bonding interface between self-adhesive resin cement to *in vitro* eroded dentin submitted to different surface treatments. The null hypotheses tested were: (1) there is no difference in the bonding strength and micromorphology of the bonding interface of a self-adhesive resin cement to sound or eroded dentin; (2) different surface treatments do not alter the bond strength and micromorphology of the bonding interface of a self-adhesive resin cement to dentin; (3) there is no difference in the bond strength of a self-adhesive resin cement to dentin 24 hours or 8 months after the bonding procedures.

## Materials and Methods

### Selection of teeth and erosion process

The research project was approved by the local Research and Ethics Committee of the Araçatuba School of Dentistry, Sao Paulo State University (#32545114.1.0000.5420), provided with an informed written consent regarding the donation of the teeth used in this study. Seventy-two freshly extracted human third molars were selected. The teeth were cleaned with curettes and kept frozen at -20°C.

The occlusal surface of the teeth was removed using a high-speed diamond saw (Isomet 2000, Buheler, Lake Bluff, IL, USA), under water cooling. After exposure of the dentin, all teeth were ground using #600 aluminum oxide abrasive papers, under water cooling, using an automatic polishing machine (Aropol E, Arotec Industria and Comercio Ltda, Cotia, SP, Brazil) for 30 s to prepare a smear layer. The teeth were divided into two groups (n = 36), sound dentin and dentin submitted to an erosive protocol [13]. This erosive protocol involved immersion of the specimens in an HCl-pepsin solution for six demineralization cycles (2 min each) per day. The demineralizing solution was prepared by dissolving 5 mg/ml NaCl in distilled water and adjusting the pH to 1.6 using HCl. Finally, 1.5 mg/ml pepsin (P-6887, pepsin from porcine gastric mucosa, Sigma–Aldrich, Seelze, Germany) was added to the HCl-pepsin solution. After each erosive process, all specimens were treated with a trypsin solution that was prepared by dissolving 2000 BAEE units/ml trypsin (T-9201, trypsin from bovine pancreas, Sigma–Aldrich, Seelze, Germany) in a mineral salt solution for 10 min. The trypsin solution contained 4.08 mM H_3_PO_4_, 20.10 mM KCl, 11.90 mM Na_2_CO_3_, and 1.98 mM CaCl_2_, buffered to a pH of 6.7.

The trypsin solution was also used for sample storage (up to 18 hours overnight) and for the composition of the slurry. After the first and last trypsin treatments, specimens were cleaned using the slurry for 15 s and an electric toothbrush (Oral-B Plak Control Ultra; Braun, Frankfurt, Germany) using a mass of 200 grams to simulate pressure during brushing.

### Preparation of the resin blocks for cementation

Seventy two blocks of TPH composite resin, color A3 (Dentsply, Petrópolis, RJ, Brazil) were manufactured in a metallic matrix (11mm in diameter and 4mm deep). The composite resin was inserted into the matrix, using a Thompson spatula, and each 2mm layer was photoactivated for 40 s using a Poly Wireless LED curing unit (Kavo, Joinville, SC, Brazil). After insertion of the last increment, a transparent polyester strip and a thin glass plate were placed on the composite to regularize the material prior to photoactivation. All of the composite resin blocks were ground using #600 aluminum oxide abrasive papers, under water cooling, in an automatic polishing machine for 30 s.

### Treatment of dentin surface prior to cementation

Both sound dentin and eroded dentin were submitted to four surface treatments, as described below (n = 9):

Control (no treatment). Blocks of TPH composite resin were cemented directly to the dentin surface using RelyX U200 self-adhesive resin cement (3M ESPE, St. Paul, MN, USA). A load of 500 grams was positioned on the block for 3 minutes, prior to photoactivation, to standardize the thickness of the resin cement. Each side was photoactivated for 40 seconds using a Poly Wireless LED curing unit (Kavo, Joinville, SC, Brazil). After cementation, the samples were stored at 37° C for 24 hours.

2% chlorhexidine gluconate (Riohex, Rioquímica, São José do Rio Preto, SP, Brazil). The samples were conditioned with 2% chlorhexidine gluconate, which was applied to the dentin surface for 60 seconds using a cotton pellet. The surface was dried with an absorbent paper. Blocks of TPH composite resin were cemented directly on the dentin surface using RelyX U200 self-adhesive resin cement (3M ESPE, St. Paul, MN, USA), as described above.

20% polyacrylic acid. The dentin surface was conditioned with 20% polyacrylic acid (Cavity conditioner, GC Corporation, Tokyo, Japan), rubbing the acid on the dentin surface for 10 seconds with a disposable applicator (KG Brush, KG Sorensen, Cotia, SP, Brazil). The prepared surface was then washed with distilled water for 20 seconds and dried using absorbent paper. Blocks of TPH composite resin were cemented directly on the dentin surface using RelyX U200 self-adhesive resin cement (3M ESPE, St. Paul, MN, USA), as described above.

EDTA. The dentin surface was conditioned for 60 seconds with 0.1M EDTA, pH 7.4 (Apothicário, Araçatuba, SP, Brazil), which was applied using a disposable applicator (KG Brush, KG Sorensen, Cotia, SP, Brazil). The surface was then washed with distilled water for 20 seconds and dried with absorbent paper. Blocks of TPH composite resin were cemented directly on the dentin surface using RelyX U200 self-adhesive resin cement (3M ESPE, St. Paul, MN, USA), as described above.

### Cutting and storage of the samples

After storage in distilled water at 37°C for 24 hours, six samples of each group were sectioned into sticks measuring approximately 1.0 x 1.0 mm x 12 mm in length using a diamond disk in a metallographic cutter (Isomet 2000, Buheler, Lake Bluff, IL, USA); eight sticks were obtained from each specimen. Half of the specimens (n = 4) were submitted to microtensile bond strength testing 24 hours after the bonding procedure, while the other sticks were stored in artificial saliva for 8 months and then tested. The artificial saliva was composed of: KH_2_PO_4_, K_2_HPO_4,_ 70% sorbitol, NaF, KCl, NaCl, MgCl, CaCl_2,_ sodium benzoate, carboxymethylcellulose and purified water (Apothicário, Araçatuba, SP, Brazil). The artificial saliva was renewed weekly.

### Evaluation of microtensile bond strength

The sticks were individually positioned in an OM 100 Odeme microtensile test machine, with a load cell of 150N (Odeme Dental Research, Luzerna, SC, Brazil) and tested at a crosshead speed of 0.7 mm per minute for microtensile bonding strength (MPa) evaluation. Sticks that showed premature failures were attributed a zero value and included in the mean of the respective group. For the microtensile testing, the extremities of the sticks were fixed to the microtensile machine using a cyanoacrylate adhesive (Super Bonder gel; Henkel Corp., Rocky Hill, Conn). The microtensile bond strength values were converted to MPa using the formula:

BS=(L/A)

Where BS = Microtensile bond strength, L = load necessary to fracture the specimen (N), A = area of the bonding interface in mm^2^.

### Evaluation of fracture pattern

After failure of the specimens, the interfaces were analyzed using optical microscopy (Leica MZ6, Leica, Wetzlar, Germany, stereomicroscope, 80x). Next, the specimens were coated with gold (Q150T, Quorum Technologies, Laughton, England) and analyzed using scanning electron microscopy (EVO LS-15, Carl Zeiss, Oberkochen, Germany) to classify the fracture pattern of the groups as: cohesive in composite resin, cohesive in the dentin surface, adhesive between resin cement and resin block, adhesive between resin cement and dentin surface, cohesive in resin cement, and mixed.

### Analysis of bonding interface morphology using confocal laser scanning microscopy (CLSM)

Three samples of each group were used for the confocal laser scanning microscopy analysis. For this analysis, 0.1% fluorescent rhodamine dye was added to the resin cement, in a proportion of 0.16 mg/g, before the bonding process [28]. In addition, another dye, fluorescein, was inserted into the pulp chamber of the teeth, 24 hours after the bonding procedures. Thereafter, the root apices were removed using a thin double-sided disc (KG Sorensen, Cotia, SP, Brazil) and water irrigation. The pulp chamber was opened through the radicular side using a #3 spherical carbide bur (KG Sorensen, Cotia, SP, Brazil) and the pulp tissue was removed using a dentin curette and an endodontic instrument. Next, the pulp chambers were irrigated using physiological saline, positioned with the occlusal surface facing downward and fixed with sticky wax in a reservoir containing distilled water to avoid dehydration. The distilled water level was kept to where it did not reach inside the pulp chamber where the solution with dye was applied. This aqueous solution was composed of fluorescein 0.1% fluorescent dye, diluted in distilled water, and was carried to the pulp chamber using a pipette. The teeth were maintained in the reservoir for at least 4 hours in order for the dye solution to reach the adhesive interface [28]. The teeth were then dried with absorbent paper and cross-sectioned using a precision diamond-cut diamond disk (Isomet 2000, Buheler, Lake Bluff, IL) to produce three slices for each specimen.

The slices were ultrasonically cleaned (Cristófoli, Campo Mourão, PR, Brazil) in distilled water for 10 minutes to remove any residue or debris. Then, the slices were examined using confocal laser scanning microscopy (Leica TCS SP2, Leica, Mannheim, Germany). A mixed helium-neon (HeNe) gas laser was used as the light source. The excitation of the light had a maximum wavelength of 543nm. The images were recorded in fluorescent mode.

An objective lens immersed in oil was used (40x, 1.25 numerical aperture). A representative area of each slice was scanned (11 sections of 2μm each). The images obtained from the mean of 11 sections were analyzed by two previously calibrated evaluators. The evaluation was performed using a double-blind method. In cases where there was divergence between the two examiners, both evaluated the image again until a consensus was reached. The criteria evaluated are in Table 1.

**Table 1:** Criteria evaluated in confocal laser scanning microscopy.

Criteria evaluated	Scores	
**Quality of dentin/cement adhesive interface [29]**	0	Absence of gaps with the interface being organized and continuous
	1	Partial presence of gaps and partial organized interface, present in approximately less than 50% of the interface
	2	Presence of gaps and disorganized interface, present in approximately more than 50% of the interface
**Formation of resin tags in dentin [29]**	0	Not detectable
	1	Few tags visible
	2	Uniform tag formation with few lateral branches
	3	Formation of long resin tags with many lateral branches
**Depth of tags [30]**	0	No tags
	1	Tags ≤ 3 μm average
	2	Tags = 3 to 8.9 μm average
	3	Tags = 9 to 15 μm average
	4	Tags ≥ 15 μm average
**Formation of hybrid layer [30]**	0	Absent
	1	Partial formation of hybrid layer up to 50% of the interface
	2	Formation of hybrid layer greater than 50%, but not the entire, interface
	3	Formation of hybrid layer along the entire interface

### Statistical analysis

The microtensile bond strength data were submitted to three-way repeated measures ANOVA (substrates, treatment groups, and time of analysis) and Tukey´s test for comparison between means (p<0.05). The dental element (n = 6 per group) was considered as the experimental unit and not the individual sticks. For the images obtained through the confocal microscopy analysis, the inter-examiner Kappa test was performed to verify the degree of agreement between the evaluators. Data were submitted to Kruskal-Wallis and Dunnet tests for statistical analysis (p<0.05).

## Results

When comparing the substrates types, Table 2 demonstrates that there were no statistically significant differences between sound and eroded dentin for the control, polyacrylic acid 20% and 0.1M EDTA groups 24h after the bonding procedures (p>0.05). However, the eroded dentin showed higher bond strength values when compared to sound dentin for the 2% chlorhexidine group (p = 0.03).

**Table 2:** Microtensile strength values, in MPa, 24 hours after adhesion, of the different types of treatments used in dentin (normal or eroded), and occurrence of premature failure of the specimens.

Group	Sound dentin	Occurrence of premature failure	Eroded dentin	Occurrence of premature failure
**Control**	7.81 ± 4.51 Ba	0/24	8.47 ± 6.00 Ba	4/24
**chlorhexidine 2%**	6.50 ± 3.3 Bb	0/24	12.43 ± 7.41 AB a	2/24
**Polyacrylic acid 20%**	14.79 ± 6.91 Aa	0/24	16.33 ± 8.90 AB a	4/24
**EDTA 0,1 M**	17.92 ± 6.61 Aa	0/24	21.35 ± 11.85 A a	2/24

Means followed by different letters (uppercase letters in the columns and lowercase letters in the rows differ statistically from each other (p<0.05).

When comparing the different treatment groups for sound dentin (Table 2), the polyacrylic acid 20% and 0.1M EDTA groups obtained higher bond strength values, with no statistical difference between them (p = 0.20). However, the control and 2% chlorhexidine groups obtained lower bond strength values when compared to the other groups (p<0.001). When considering eroded dentin, the 0.1M EDTA group showed higher bond strength values with a statistically significant difference only for the control group (p = 0.002).

After 8 months of storage (Table 3), the present results showed that there was no statistically significant difference between the two substrates for all experimental groups (p>0.05). In the comparison of different treatments for sound dentin, the 0.1M EDTA group showed the highest values of bond strength, with a statistically significant difference when compared to the control and 2% chlorhexidine groups (p<0.05). The 20% polyacrylic acid group did not show a significant difference when compared to the 0.1M EDTA and 2% chlorhexidine groups (p>0.05). For eroded dentin, there was no statistically significant difference among the surface treatments, including the control group (p>0.05).

**Table 3:** Microtensile strength values, in MPa, 8 months after adhesion, of the different types of treatments used in dentin (normal or eroded), and occurrence of premature failure of the specimens.

Group	Sound dentin	Occurrence of premature failure	Eroded dentin	Occurrence of premature failure
**Control**	5.24 ± 3.67 Ca	4/24	8.50 ± 3.43 Aa	4/24
**chlorhexidine 2%**	7.15 ± 4.61BCa	1/24	13.90 ± 8.60 Aa	4/24
**Polyacrylic acid 20%**	12.14 ± 8.94 Aba	3/24	11.57 ± 6.50 A a	2/24
**EDTA 0,1 M**	19.67 ± 5.98 Aa	0/24	16.97 ± 7.12 Aa	0/24

Means followed by different letters (uppercase letters in the columns and lowercase letters in the rows differ statistically from each other (p<0.05).

In the comparison of the periods of analysis, higher values of bonding strength were generally found after 24 h storage when compared to the analysis after 8 months storage (p = 0.04). However, for each group separately, there were no statistically significant differences among all the experimental groups (p>0.05).

**Fig 1:**
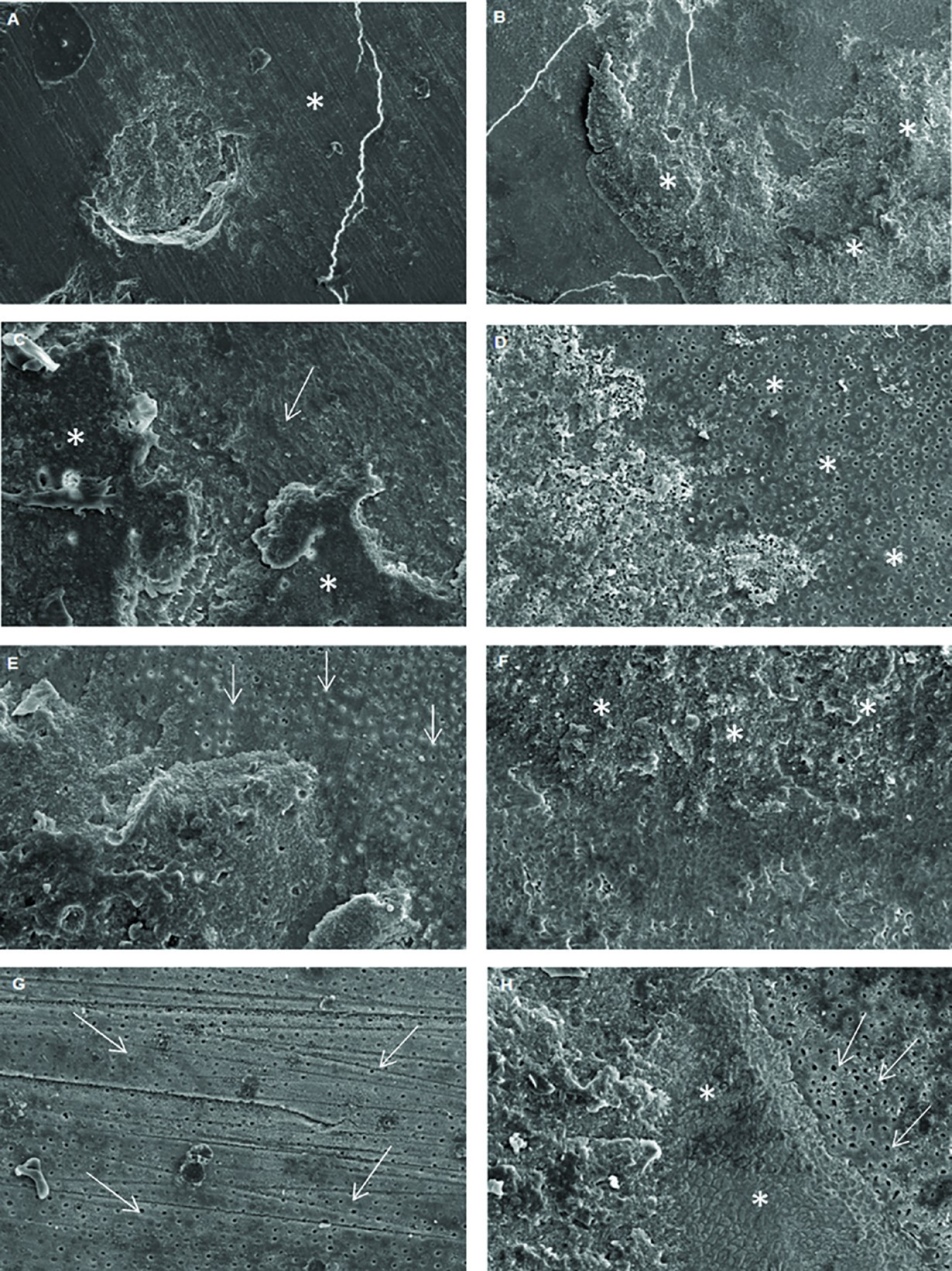
Scanning electron microscopy analysis of the different groups in sound and eroded dentin, 24 hours after the adhesive process. (A) Representative specimen of the control group–sound dentin. Underlying dentin without exposure of the dentinal tubules (asterisk). (B) Representative specimen of the control group–eroded dentin, where it is possible to observe a greater bonding of the cement to the eroded substrate (asterisks). (C) Representative specimen 2% chlorhexidine sound dentin, it is to observe the presence of the resin cement (asterisks) on the smear layer (arrow). (D) Representative specimen 2% chlorhexidine eroded dentin, there is a remarkable exposure of the dentinal tubules compared to sound dentin (arrows). (E) Representative specimen 20% polyacrylic acid sound dentin, presence of the dentinal tubules partially occluded by the smear plug (arrows). (F) Representative specimen 20% polyacrylic acid eroded dentin, evidencing the presence of cement on the dentin (asterisks). (G) Representative specimen 0.1 M EDTA–sound dentin, it is noted that both the smear layer and the smear plug were removed, with great exposure of the dentinal tubules (arrows). (H) Representative specimen 0.1 M EDTA–eroded dentin, the presence of cement on the left with adjacent smear layer (asterisks), and evident increase in the opening of the dentinal tubules in the regions of exposed dentin (arrows), compared to the sound dentin of the same group. Magnification 1000x.

**Fig 2:**
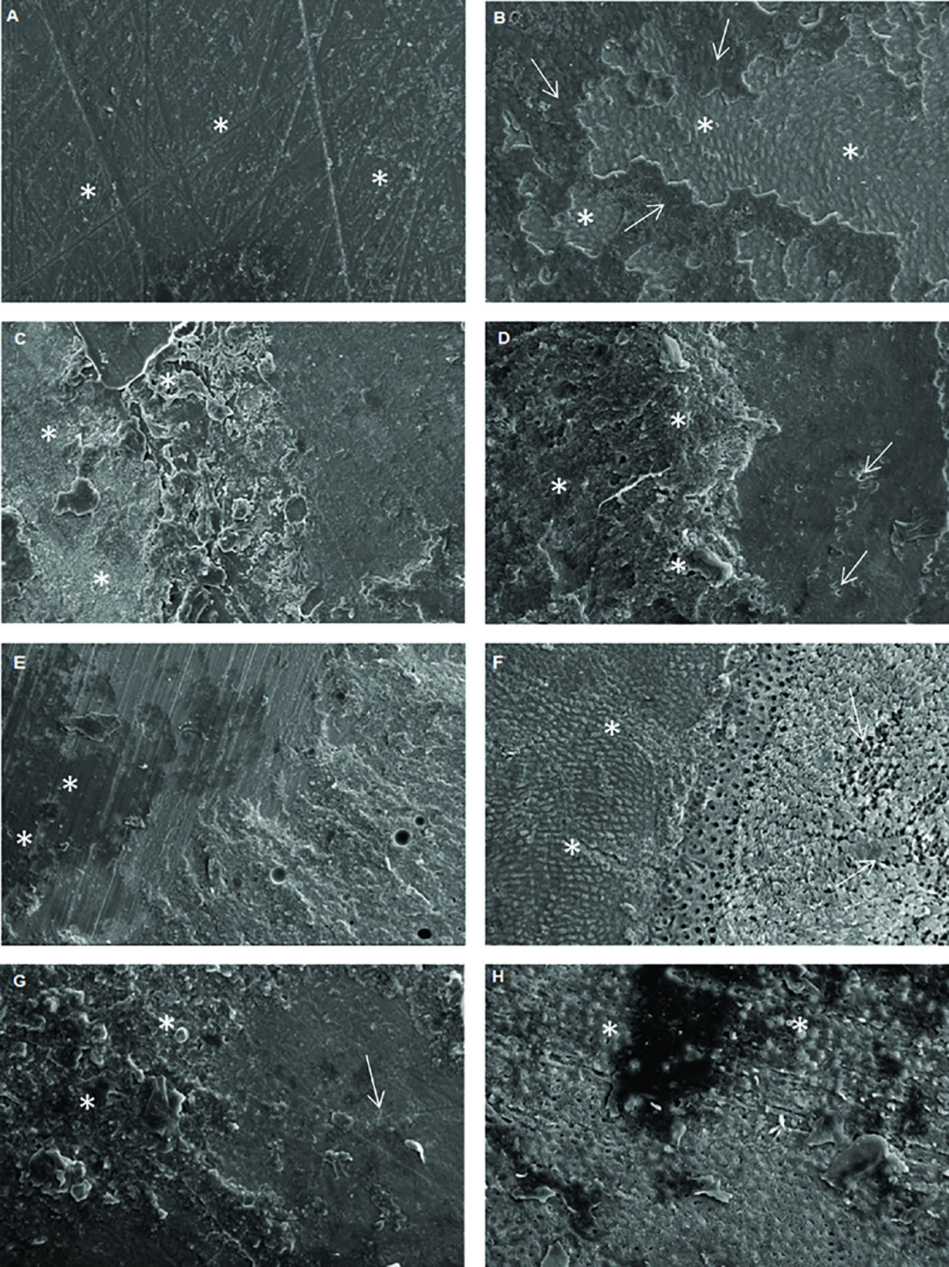
Scanning electron microscopy analysis of the different groups in sound and eroded dentin, 8 months after the adhesive process. (A) Control group–sound dentin. Few or no presence of cement on the dentin, without exposure of the dentinal tubules (asterisks). (B) Control group–eroded dentin, showing the presence of mixed failure, with smear layer areas (asterisks) surrounded by resin cement (arrows). (C) 2% Chlorhexidine group–sound dentin. Presence of the resin cement on the dentin surface (asterisks), without exposure of the dentinal tubules. (D) 2% Chlorhexidine group–eroded dentin. Presence of the resin cement on the dentin surface (asterisks), whit few exposure of the dentinal tubules (arrows). (E) 20% Polyacrylic acid group–sound dentin. Few exposure of the dentinal tubules (asterisks). (F) 20% Polyacrylic acid group–eroded dentin. It is evidenced the presence of resinous material on the smear layer (asterisks) and many dentinal tubules exposed on the surface, with exposure of the collagen fibers (arrows); most of the exposed tubules are obliterated. (G) 0.1 M EDTA group–sound dentin, evidencing the presence of resin cement (asterisks), with few exposure of the dentinal tubules (arrow). (H) 0.1 M EDTA group–eroded dentin, evidencing the presence of a few exposed tubules on the surface and others filled by the resin cement (asterisks). Magnification 1000x.

**Fig 3:**
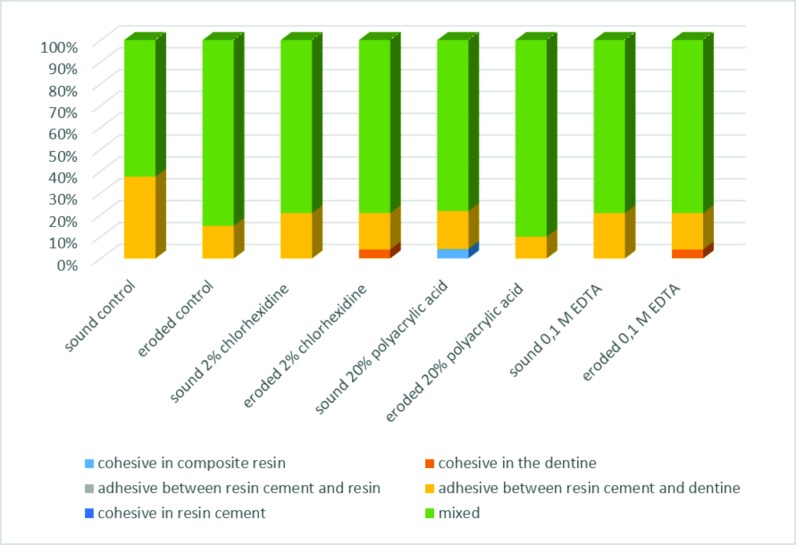
Incidence (%) of failures mode among treatment groups, 24 hours after the adhesion process.

**Fig 4:**
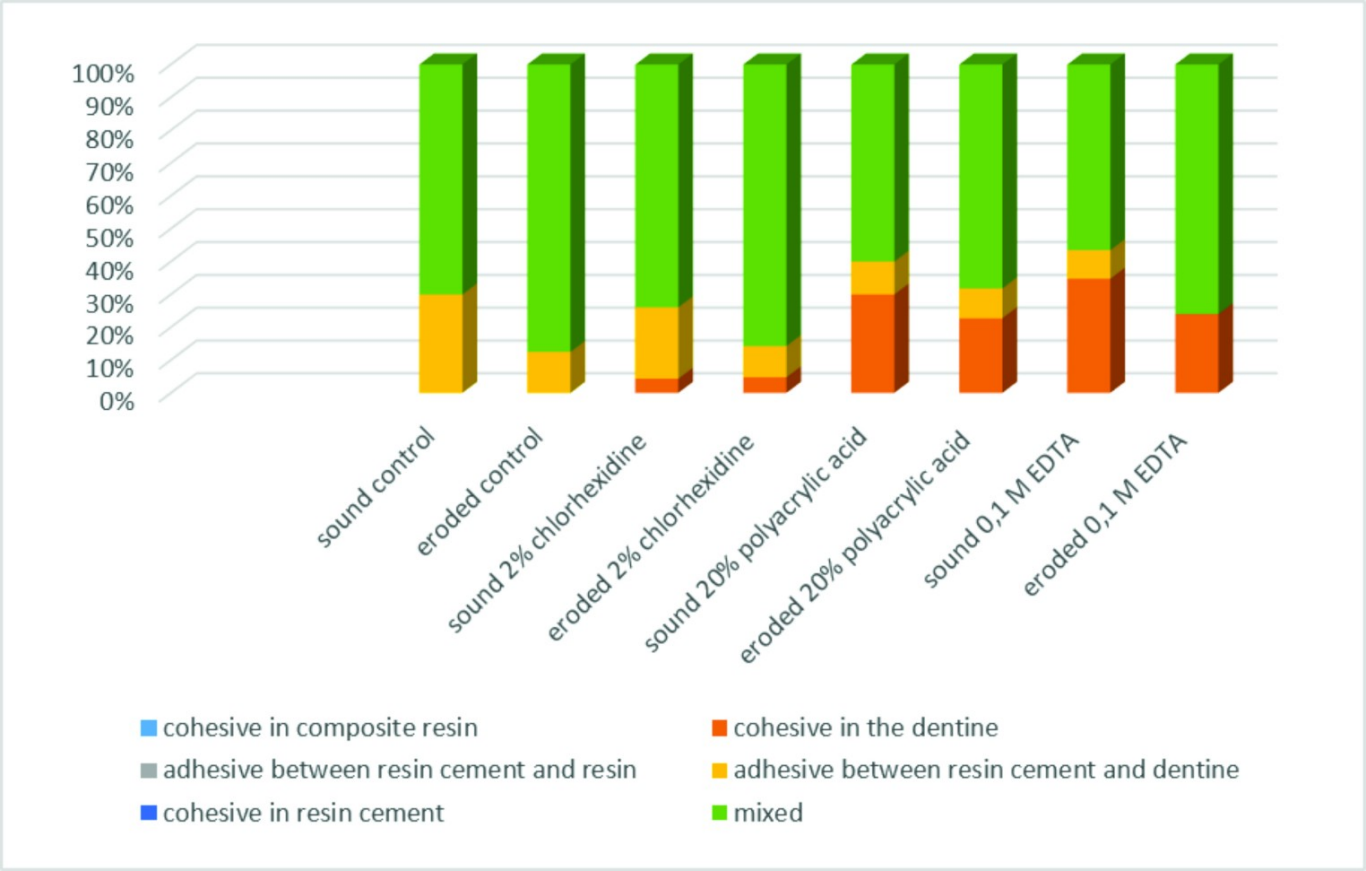
Incidence (%) of failures mode among treatment groups, 8 months after the adhesion process.

In relation to the number of premature failures, eroded dentin presented a higher number of premature failures when compared to sound dentin, especially 24 hours after the bonding procedures (Tables 2 and 3; Figs 1 and 2). Among different treatments, the lowest number of premature failures were found for the 0.1M EDTA group, regardless of the storage time. There was a predominance of mixed-type failures (adhesive and cohesive failure in dentin), Figs 3 and 4, for both of the dentin substrates. However, a considerable number of cohesive in dentin failures (failure of the tooth substrate), especially for the 20% polyacrylic acid and 0.1M EDTA groups, were found after 8 months of storage (Fig 4).

**Fig 5:**
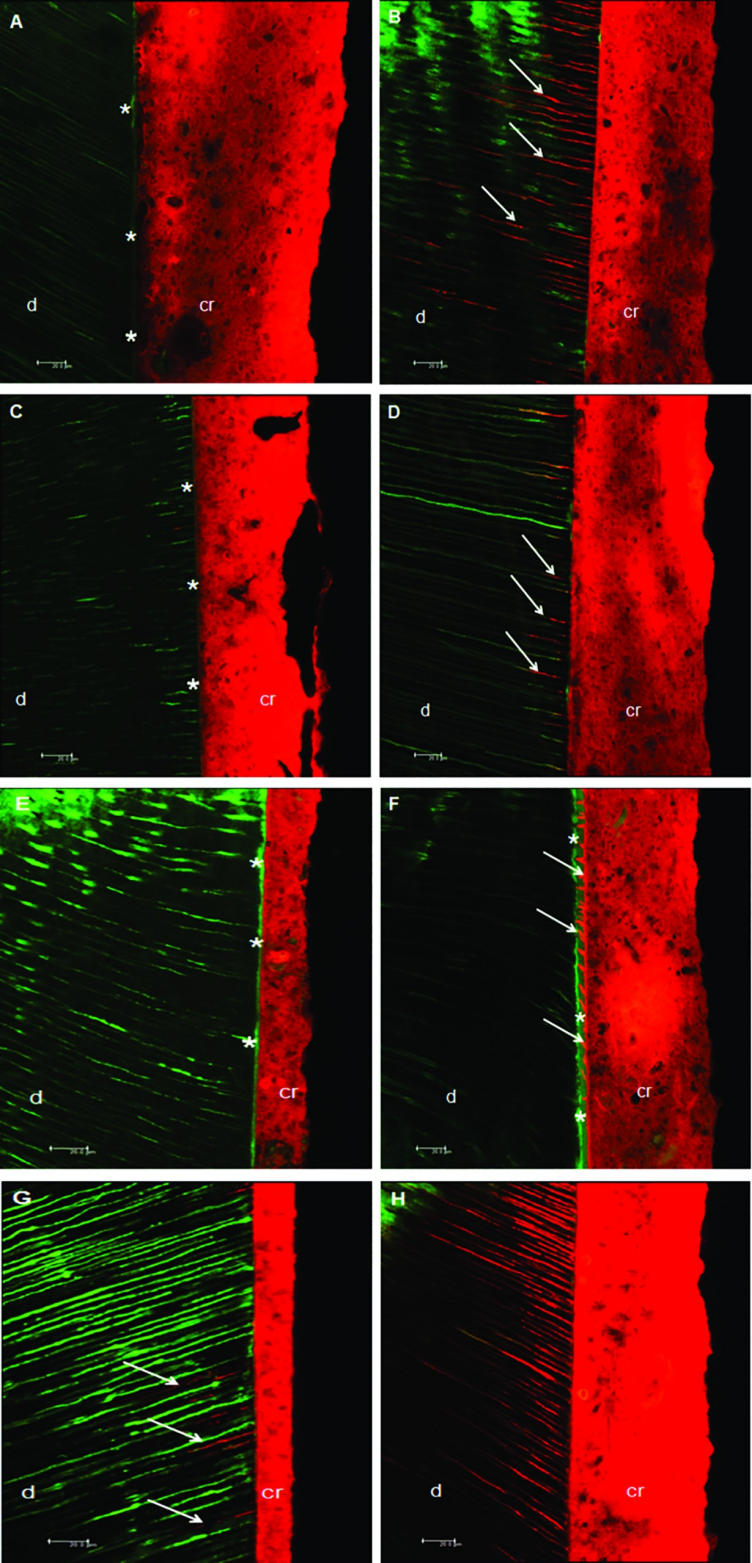
Analysis in confocal microscopy of sound and eroded dentin submitted to different surface treatments. (A) Control group–sound dentin. It is possible to observe a continuous interface, without cracks (asterisks) and without the formation of tags. (B) Control group–eroded dentin. Adhesive interface without hydrid layer formation, however, an intense formation of resin tags (arrows) was observed in relation to the sound dentin. (C) 2% Chlorhexidine group–sound dentin. Note the absence of cracks in the adhesive interface as well as hybrid layer formation (asterisks). (D) 2% Chlorhexidine group–eroded dentin. Absence of cracks in the adhesive interface, with formation of some visible tags (arrows). (E) 20% Polyacrylic acid group–sound dentin. Pronounced hybrid layer formation throughout the adhesive interface (asterisks). (F) 20% Polyacrylic acid group–eroded dentin. Hybrid layer formation throughout the adhesive interface, with increased thickness compared to sound dentin (asterisks) and tag formation ≤ 3 μm in almost all interface (arrows). (G) 0.1 M EDTA group–sound dentin. Regarding the quality of the adhesive interface, it is possible to note the absence of cracks, with the formation of some resin tags measuring from 9 to 15 μm (arrows). (H) 0.1 M EDTA group–eroded dentin. Absence of cracks in the adhesive interface, without hybrid layer, but with the very dense formation of resin tags as well as in depth. *d = dentin / *rc = resin cement. Asterisks: hybrid layer formation; arrows: resin tags formation.

The results of the bonded interface, when examined under confocal microscopy, is presented in Table 4 and Fig 5. A Kappa inter-examiner test was performed and a value of 0.67 was found, which is considered a substantial value for this type of evaluation. In relation to the quality of the adhesive interface, there were no statistical differences among the groups for sound and eroded dentin (p>0.05), with a predominance of score 0, with no cracks in the adhesive interface and which was considered continuous and well organized. For the formation of resin tags in sound dentin, different groups presented a higher number of score 0 (no tag formation). However, for eroded dentin, there was a tendency for higher scores for resin tag formation, especially for the 0.1M EDTA group. There was a statistical difference between the sound and eroded dentin only for the control group (p<0.05). In relation to the depth of tags, the same phenomenon was observed, with a tendency of greater depth of tags for the eroded dentin when compared to sound dentin, which was statistically significant for the control and 2% chlorhexidine groups (p<0.05).

**Table 4:** Qualitative (scores) in confocal microscopy.

Parameters		Quality of the adhesive interface dentin/cement				Dentin tag formation					Depth of tag penetration						Hybrid layer formation				
Scores		0	1	2		0	1	2	3		0	1	2	3	4		0	1	2	3	
Control	S	6	2	˗	A	6	2	˗	˗	B	7	˗	˗	1	˗	B	2	1	˗	5	AB
	E	8	˗	˗	A	˗	˗	˗	8	A	˗	˗	˗	˗	8	A	7	1	˗	˗	B
Chlorhexidine 2%	S	6	2	˗	A	6	2	˗	˗	B	6	˗	2	˗	˗	B	1	˗	2	5	A
	E	8	˗	˗	A	˗	5	1	2	AB	˗	˗	2	˗	6	A	4	2	2	˗	AB
Polyacrylic acid 20%	S	5	2	1	A	5	2	1	˗	B	4	4	˗	˗	˗	B	4	1	˗	3	AB
	E	3	2	3	A	2	4	2	˗	AB	2	4	1	1	˗	AB	1	3	˗	4	AB
EDTA 0.1M	S	8	˗	˗	A	˗	5	2	1	AB	˗	˗	1	2	5	AB	8	˗	˗	˗	B
	E	8	˗	˗	A	˗	1	˗	7	A	˗	1	˗	˗	7	A	4	2	1	1	AB

Means followed by different letters in the columns differ statistically from each other (p<0.05). S: sound; E: eroded

With regards to the formation of the hybrid layer for the sound dentin, the control and 2% chlorhexidine groups presented higher scores in comparison to other groups, with a predominance of score 3, indicating hybrid layer formation across the adhesive interface. The hybrid layer was more evident in the 20% polyacrylic acid group for eroded dentin. There was no statistical difference between sound and eroded dentin for all the groups (p>0.05).

## Discussion

The microtensile bond test allows for the measurement of small interfaces and the ability to obtain several specimens from the same tooth [31,32,33]. Moreover, is more sensitive to surface defects [33,34]. In the present study, eight sticks per tooth were obtained, four of which were used for the bond strength testing 24h after the bonding procedure and the other four tested 8 months after storage. Sticks that presented premature fracture were assigned a value of zero for bond strength (Tables 2 and 3) [35]; therefore, the tooth was used as the experimental unit. In relation to the fracture patterns observed, the mixed-type failure was the most common among the groups for both sound and eroded dentin, followed by adhesive-type failure between dentin/cement and cohesive-type failure in dentin (Figs 3 and 4), corroborating with another study [34], indicating that the mixed-type and adhesive-type failures are the most common types when self-adhesive cements are used.

Analysis of the micromechanical interaction of the adhesive interface was performed using confocal microscopy. This method evaluates the micropermeability and the sealing ability of the resin tags at the adhesive interface by using fluorescent dyes in the dental substrate and/ or resinous material [36], allowing for the evaluation of the subsurface. This is a non-destructive method which offers more details regarding the identification of the structures that constitute the adhesive interface [29] with less artefacts and decreased halo formation in the image when compared to scanning electronic microscopy [36].

Based on the results of the study, there was a significant difference in the bond strength between sound and eroded dentin when using 2% chlorhexidine, 24 hours after the bonding procedures, causing the first null hypothesis of the study to be rejected. The naturally eroded dentin generally presents reactionary dentin with a mineral deposition occluding the dentin tubules, forming a hypermineralized layer [37]. These could contribute to the decrease in bond strength for this substrate, especially when self-adhesive resin materials are used, since these materials demineralize the substrate in a milder manner when compared to conventional resin cements [21,22]. When comparing naturally eroded dentin to *in vitro* eroded dentin, it is believed that the latter could present a much more accentuated demineralization than *in vivo* lesions, with greater exposure of the collagen network and possible greater mechanical interaction with resin monomers when compared to a natural lesion [38]. However, the present study used the *in vitro* erosive protocol that utilizes pepsin and trypsin enzymes, which are present in the gastrointestinal tract and contact the dental tissues during gastroesophageal reflux and / or vomiting [6]. Pepsin appears to act in the degradation of the dentin collagen matrix, without accentuating its mineral loss. On the other hand, trypsin would have its collagenolytic activity favored by protein denaturation caused by pepsin, acting much more in the modification of the collagen ultra-structure, destabilizing it so that pepsin can bind more effectively to this molecule; thereby increasing the degradation of the organic matrix [6]. These enzymes appear to be much more related to the modification or partial degradation of the organic matrix than to the substantial loss of mineral tissue, and do not appear to have contributed to a change in bond strength values when compared to sound dentin, especially for the control, chlorhexidine, and EDTA groups.

This study used a self-adhesive resin cement, which contains acid ester monomers to demineralize the dentin surface while also infiltrating it. Other studies utilized different dentin surface treatments before the luting procedures, as some substances could optimize the penetration of the material into dentin [19,26,39]. Based on the results obtained in this study, the different surface treatments showed an influence in both bond strength and micromechanical interaction of the adhesive interface, causing the second null hypothesis of this study to be rejected. Chlorhexidine is a chemical agent that has been widely studied in dentin surface treatments. Since chlorhexidine is an inhibitor of collagenous activity, it contributes to the structural integrity of the hybrid layer [19,24,25,26]. In the confocal microscopy analysis, there was greater formation or preservation of the hybrid layer for this group, especially in sound dentin (Table 4, Fig 5C). On the other hand, this substance has an affinity for the phosphate groups contained in dentin, making the bonding procedure more difficult, since it minimizes the remaining apatite of the organic matrix which could chemically bind to the acid monomers present in the resin cement [19]. Moreover, its affinity for calcium ions could also decrease the mineral available for cement bonding [27]; these facts may have contributed to the lower bond strength values obtained for this group in sound dentin. In contrast, the higher values of bond strength when using chlorhexidine in eroded dentin were notable. This may be related to the high affinity of chlorhexidine for the demineralized dentin, which may be up to 80% greater than its bond to mineralized dentin [24,26]. Chlorhexidine 2% was used because most of the studies use this concentration, according to the review by Montagner et al. 2014 [40]. Francisconi et al. 2015a [41] used chlorhexidine at 24h, 6 months and 1 year and noted that for the 6-month period there was a maintenance in the bond strength values for both normal and eroded teeth. In addition, Francisconi et al. 2015b [42] compared 0.004% and 2% chlorhexidine concentrations and concluded that the concentration of 2% showed better performance in the bond strength. Frassetto et al. 2016 [24] related that chlorhexidine may loss its inhibitory effect of MMPs in the presence of calcium chloride, so concentrations as low as 0.5 or 1% would be insufficient to inhibit MMPs, requiring a concentration of 2%. Furthermore, collagen and MMPs may compete with for chlorhexidine binding, requiring the use of relatively high concentrations. Thus, it is speculated that chlorhexidine may have contributed to the maintenance of the collagen network that was infiltrated by the resinous monomers in greater intensity than to sound dentin, increasing the bond strength values for eroded dentin (Table 2). Scanning electron microscopy noted that the application of chlorhexidine in previously eroded dentin promoted greater exposure of the dentinal tubules when compared to the sound dentin group (Fig 1C) after 24 hours of storage.

Polyacrylic acid was also used as a surface treatment in the current study. Sound dentin previously conditioned with polyacrylic acid showed higher values of bond strength when compared to the control group (Tables 2 and 3). Some studies, in which 25% or 10% polyacrylic acid was used, showed higher values of bond strength in relation to the control group [23,39,43]. This increase in bond strength values could be attributed to the hybrid layer formation [44] as can be seen in some confocal microscopy images (Fig 5E and 5F). Moreover, the acid partially removed the smear layer, resulting in a surface filled with calcium and phosphate ions that could react with the acidic monomers present in the resin cement, resulting in a chemical bond [23,39,43]. Polyacrylic acid can also completely remove the smear layer, facilitating the penetration of the resin cement between the collagen fibers to form a hybrid complex [23]. In addition, the carboxyl ions present in the polyacrylic acid have the ability to form multiple hydrogen bonds with the dentin substrate [43], which could explain the highest bond strength values obtained for this group.

Another chemical agent utilized in the current study was EDTA, which has calcium ion chelating properties with an inhibitory action on MMPs [25]. The MMPs are present in the natural constitution of dentin; when the pH decreases, as with the application of the acid monomers contained in the resin cements, these enzymes that act in hydrolytic degradation of the collagen fibers are activated [45]. Thus, the application of EDTA could contribute to the preservation of the mechanical properties of the adhesive interface, since it would maintain the mechanical bonding between the collagen matrix and the resinous monomers [26,46]. In addition, EDTA is considered a moderate acting acid [47], it acts by dissolving the mineral phase of the dentin, without causing deproteinization of the collagen fibers, thus maintaining its original structure [39,47,48]. The removal of the smear layer and smear plug by EDTA (Fig 1G and 1H) reinforces micromechanical retention, since the collagen fibers remain unchanged and unaltered with regards to the mineral content, thus, the chemical bonding of the calcium ions with the monomers of the resinous material is improved [48]. In the present study, higher bond strength values were generally obtained for the group with EDTA used as a dentin pre-treatment (Tables 2 and 3), which has been observed in other studies [39,46].

The results showed that the bond strength values were generally higher after 24 hours of storage when compared to evaluation after 8 months, thus rejecting the third null hypothesis of the study. However, when the experimental groups (sound and eroded dentin) were individually analyzed, there was no significant statistical difference between times of analysis for all studied groups. Although the specimens had a reduced size (around 1mm^2^), which theoretically would be more susceptible to water diffusion along the interface and accelerating hydrolytic degradation [49], storage in artificial saliva for 8 months seemed to not have caused significant degradation in the adhesive interface when the groups were analyzed separately, likely due to the smaller number of samples analyzed within each group when compared to the general analysis. Aguiar et al. 2014 [49] noticed significantly decreased bond strength values for the self-adhesive resin cements after 2 years of aging. Leme et al. 2015 [50] showed degradation of the dentin-adhesive interface after 30 months of storage in artificial saliva. Ren et at. 2018 [51] comparing etch & rise, self-etch and self adhesives resin cements in storage times of 24 hours and 2 years in water, concluded that there was a significant decrease in bond strength for the self-etch and self-adhesives materials. Almeida et al. 2018 [52] compared the bond strength between self-adhesive resin cements at 24h, 6 month and 1 year of water storage times and found that at the 6-month, the values remained stable for most of the cements tested. In the same way, Simões et al. 2016 [53] also verified a stability in the bond strength values for the resin cement in 6 months of storage. Blumer et al. [31] compared some artificial aging methods for resin cements and concluded that thermal cycling acted more substantially for adhesive interface degradation due to the abrupt temperature changes which promoted dimensional changes in both the organic matrix and filler of the cement, facilitating the penetration of water and accentuating the degradation of the adhesive interface [31].

In the confocal microscopy images (Fig 5), the adhesive interface for both sound and eroded dentin was found to have a good interface seal with the formation of few cracks, and in many specimens there were no visible cracks. The eroded dentin showed an increase in both density and depth of resin tag formation when compared to sound dentin for all experimental groups (Table 4, Fig 5). It is speculated that the erosive protocol may have superficially removed the smear layer and smear plug, thus contributing to a greater penetration of the resin cement. The depth of penetration of the tags was also greater for the eroded dentin, since the erosive process seems to have contributed to a partial mineral dissolution of the substrate, allowing for greater penetration of the cement into the organic matrix. In relation to the formation of the hybrid layer, the groups with 20% polyacrylic acid presented the formation of a pronounced hybrid layer in most of the specimens, as shown in Fig 5E and 5F.

The present study has some limitations, including the poor effect of aging on bonding strength values and the difficulty of standardizing the age of the teeth used, since the dentin is an age-dependent and changeable substrate. Especially in relation to biochemical alteration, old dentin has more tubule obliteration, because there is a formation of reactionary dentin, which may presented lower bond strength due to this phenomenon. Thus, it is known that the age difference between dentin may contribute to possible variations in the bond strength [37]. According to Ivancik et al. 2012 [54], which compared young and old groups of third molar teeth to the fatigue crack growth resistance, concluded that older age and perpendicular orientation tubules may interfere negatively, increasing the chance of fractures. The results obtained seem to contribute to the understanding of how the adhesive process would occur in previously eroded dentinal tissue. Other studies, with a main focus on the biochemical alterations that would occur in the dentin surface, as well as longer time evaluations, are necessary to complement these findings.

## Conclusion

The previously eroded dentin presented a considerable increase in the density and depth of resin tags in relation to sound dentin, although little difference was found in the microtensile bond strength values when comparing the two substrates. The different surface treatments generally promoted an increase in the microtensile bond strength when compared to the control group. The storage of the samples in artificial saliva for 8 months seems to be insufficient to cause significant degradation of the adhesive interface.
